# PpMYC2 and PpJAM2/3 antagonistically regulate lignin synthesis to cope with the disease in peach fruit

**DOI:** 10.1111/pbi.70177

**Published:** 2025-06-04

**Authors:** Qian Li, Yi Chen, Yingying Wei, Shu Jiang, Jianfen Ye, Jiahui Chen, Feng Xu, Xingfeng Shao

**Affiliations:** ^1^ State Key Laboratory for Quality and Safety of Agro‐products, Zhejiang Key Laboratory of Intelligent Food Logistic and Processing, Zhejiang‐Malaysia Joint Research Laboratory for Agricultural Product Processing and Nutrition, College of Food Science and Engineering Ningbo University Ningbo China

**Keywords:** *Prunus persica*, transcription factor, lignin synthesis, *Monilinia fructicola*, competitive binding

## Abstract

Transcription factors MYC2 and JAMs play a crucial role in regulating disease resistance in model plants. However, their regulatory mechanisms of disease resistance in non‐model plants, particularly in postharvest fruits, remain largely unknown. In this study, we observed that *PpMYC2* expression was up‐regulated in peach fruit following infection by *Monilinia fructicola*, while the expressions of *PpJAM2/3* were down‐regulated. Furthermore, we found that PpMYC2 positively regulated the resistance against *M. fructicola*, whereas PpJAM2/3 negatively regulated it. Through a combined DNA affinity purification and RNA sequencing analysis for PpMYC2, we identified lignin synthesis genes (*PpPAL1*, *PpC4H*, *Pp4CL1*, *PpCSE* and *PpCCoAOMT1*) as candidate target genes. Subsequent assays, including dual‐luciferase reporter assay, transient overexpression and silencing assays, electrophoretic mobility shift assay and yeast one‐hybrid assay demonstrated that PpMYC2 activated the transcription of these five genes by binding to their promoters, promoting lignin accumulation. Conversely, PpJAM2 inhibited the transcription of *PpC4H* and *PpCSE*, while PpJAM3 inhibited *Pp4CL1* and *PpCCoAOMT1*. Additionally, PpJAM2 or PpJAM3 interfered with PpMYC2's activation of their common target genes by competitively binding to the promoters. In conclusion, when peach fruit is infected with *M. fructicola*, up‐regulation of *PpMYC2* promotes lignin synthesis, while down‐regulation of *PpJAM2/3* reduces their inhibitory effects, ultimately resulting in lignin accumulation to combat the disease infection. Our study provides new insights into the molecular mechanisms of disease response in postharvest peach fruit.

## Introduction

To cope with biotic and abiotic stresses, plants evolve sophisticated regulatory strategies, among which transcription factors are crucial for regulating plant defence responses. Transcription factors are divided into different families according to conserved domains, such as bHLH, WRKY, NAC, bZIP and AP2/ERF. The bHLH transcription factor, one of the largest families, is characterized by two conserved domains: a basic DNA‐binding region and a helix–loop–helix region (de Martin *et al*., 2021). MYC2 transcription factor, a member of the bHLH family, possesses the conserved bHLH domain (Sasaki *et al*., 2001). MYC2 transcription factor is a key component of the hormone regulatory network with jasmonic acid (JA) as the core, participating in regulating many biological processes, such as the synthesis of secondary metabolites, biotic and abiotic stresses and plant growth and development (Boter *et al*., 2004; Luo *et al*., 2023; Shoji and Hashimoto, 2011). In the absence of stimulation, the jasmonate ZIM‐domain (JAZ) protein interacts with MYC2, inhibiting MYC2's regulation of JA‐response genes. Under external stimulation, JA is rapidly synthesized, leading to proteasome‐dependent degradation of JAZ repressor proteins, and then, MYC2 is released, allowing it to regulate the expression of JA‐response genes (Kazan and Manners, 2013; Thines *et al*., 2007).

Currently, the studies on the role of MYC2 in disease regulation are mainly concentrated on model plants. In tomato, SlMYC2 activated JA‐mediated plant immunity through a hierarchical transcriptional cascade (Du *et al*., 2017). Min *et al*. (2020) reported that SlMYC2 participated in MeJA‐induced resistance of tomato fruit to *Botrytis cinerea* through the regulation of defence enzyme activities, the expression of pathogenesis‐related protein (PR) genes, as well as the production of α‐tomatine and phenylpropanoids. In *Arabidopsis thaliana* (Arabidopsis), AtMYC2 participated in triggering early‐stage defence responses during the systemic resistance induced by rhizobacteria (Pozo *et al*., 2008). In rice, the overexpression of *OsMYC2* enhanced the resistance to bacterial blight, whereas the knocking out of *OsMYC2* significantly reduced the JA‐dependent activation of numerous defence‐related genes and defensive compounds (Ogawa *et al*., 2017; Uji *et al*., 2016). JA‐ASSOCIATED MYC2‐like (JAM) transcription factor, which belongs to the family of bHLH transcription factors. Its expression pattern was initially found to be closely related to the expression pattern of JA‐response genes. In Arabidopsis, the root and anthocyanin phenotype in *jam1/2/3* triple mutants was attenuated by *myc2*, suggesting that MYC2 and JAMs function antagonistically (Sasaki‐Sekimoto *et al*., 2013). AtJAM2 (AtbHLH13) negatively regulated the defence against *B. cinerea* and *Spodoptera exigua* (Huang *et al*., 2018). In tomato, SlMTB1/2/3, homologues of the Arabidopsis JAM proteins, negatively regulated plant resistance to grey mould infection but played an active role in the resistance to *Pst* DC3000 infection (Liu *et al*., 2019). Additionally, MTBs inhibited JA‐mediated transcriptional responses by interfering with the function of the MYC2‐MED25 transcriptional activation complex. Except for tomato fruit, the regulatory effect of MYC2 on disease resistance in other fruits has not yet been reported, while the role of JAMs on disease resistance of postharvest fruits remains unclear.

Lignin is a key structural component of plant cell walls. Its synthesis involves the generation of phenylpropanoid compounds and lignin monomers, which are ultimately polymerized to form lignin (Zhu *et al*., 2020). When the plant is invaded by pathogens, the cells in the infected area and its surroundings rapidly lignify against pathogens, restricting the spread of pathogens within the plant (Ma *et al*., 2018). Lignin can bind the toxins of pathogens, reducing their activity and damage to plant cells (Karmanov *et al*., 2023). Additionally, lignin synthesis activates plant defence signalling and up‐regulates defence genes. Transcription factors participate in lignin synthesis by regulating the expression of lignin synthesis genes (Liu *et al*., 2018; Wang *et al*., 2018). CsAP2L and CsERF1B transcription factors up‐regulated the expressions of lignin synthesis genes and promoted lignin accumulation, positively regulating citrus fruit resistance to *Penicillum digitorum* (Chen *et al*., 2023; Li *et al*., 2023a). OsNAC055 modulates the lignin biosynthesis in rice straw by activating *OsCCR10* and *OsCAD2* (Liu *et al*., 2022). Lignin synthesis is also regulated by many plant hormones, among which JA and its related molecules are effective stimulants for lignin biosynthesis (Liu *et al*., 2023). Xu *et al*. (2024) reported that wounding stimulated lignin deposition by initiating a transient JA signal, which AtMYC2 subsequently transmitted to abscisic acid (ABA) signalling to maintain lignin biosynthesis by increasing *AtRAP2.6* expression, promoting the later wound healing process. In rice, the overexpression of the JA‐responsive gene *OsbHLH034* led to enhanced resistance to bacterial blight by promoting lignin biosynthesis (Onohata and Gomi, 2020). Additionally, exogenously applied methyl jasmonate (MeJA) in potato plant up‐regulated the expression of lignin synthesis genes (Yang *et al*., 2022).

Brown rot, caused by the fungal pathogen *Monilinia fructicola*, is a prevalent and destructive disease impacting various stone fruits globally (Cheng *et al*., 2023). Peach fruit is one of the most representative fruits of the *Rosaceae* family and is beloved by consumers due to its delightful flavour and high nutritional value (Xin *et al*., 2018). According to the previous survey results, *M. fructicola* could cause 30–80% of peach fruit to rot, and it poses a major threat not only by causing substantial economic losses in the peach industry but also by affecting a range of other fruits, such as plums, sweet cherries, apricots and dates, making it a serious concern for the entire fruit sector (Huang *et al*., 2024). Therefore, there is a necessity to explore the underlying mechanisms of disease development at the molecular level, which is crucial for developing more effective, targeted and environmentally friendly control strategies.

In the present study, we investigated the roles of PpMYC2 and PpJAM2/3 in peach fruit disease resistance. Through genome‐wide binding site analysis and RNA sequencing (RNA‐seq), we identified the candidate target genes regulated by PpMYC2 that were associated with disease resistance in peach fruit. Moreover, we examined the regulatory effects of PpMYC2 and PpJAM2/3 on these genes, as well as the regulatory relationship between PpMYC2 and PpJAM2/3 on their common target genes. Overall, our findings will provide valuable insights into the transcriptional regulatory networks involved in fruit postharvest diseases.

## Results

### Phylogenetic analysis, multiple sequence alignment and subcellular localization of PpMYC2 and PpJAM2/3

Phylogenetic tree analysis showed that PpMYC2 was most closely related to SlMYC2, followed by AtMYC2. PpJAM2 and PpJAM3 were most closely related to AtJAM2 and SlMTB3, respectively, followed by AtMTB1/2 and AtJAM3 (Figure S1). Multiple sequence alignment analysis revealed that PpMYC2 and PpJAM2/3 all possess a basic region at the N‐terminal and a HLH region at the C‐terminal (Figure S2), which is characteristic of a bHLH transcription factor. The subcellular localization of PpMYC2 and PpJAM2/3 was determined in tobacco leaves via *Agrobacterium*‐mediated transformation. As shown in Figure S3, the green fluorescent protein (GFP) fluorescence signals of the control were dispersed evenly around the nucleus and membrane. However, the GFP fluorescence signals of PpMYC2‐GFP, PpJAM2‐GFP and PpJAM3‐GFP were located exclusively in the nucleus, suggesting that these three transcription factors functioned as nuclear proteins.

### Different expression patterns of *PpMYC2* and *PpJAM2/3* in peach fruit infected with *M. fructicola*

**Figure 1:**
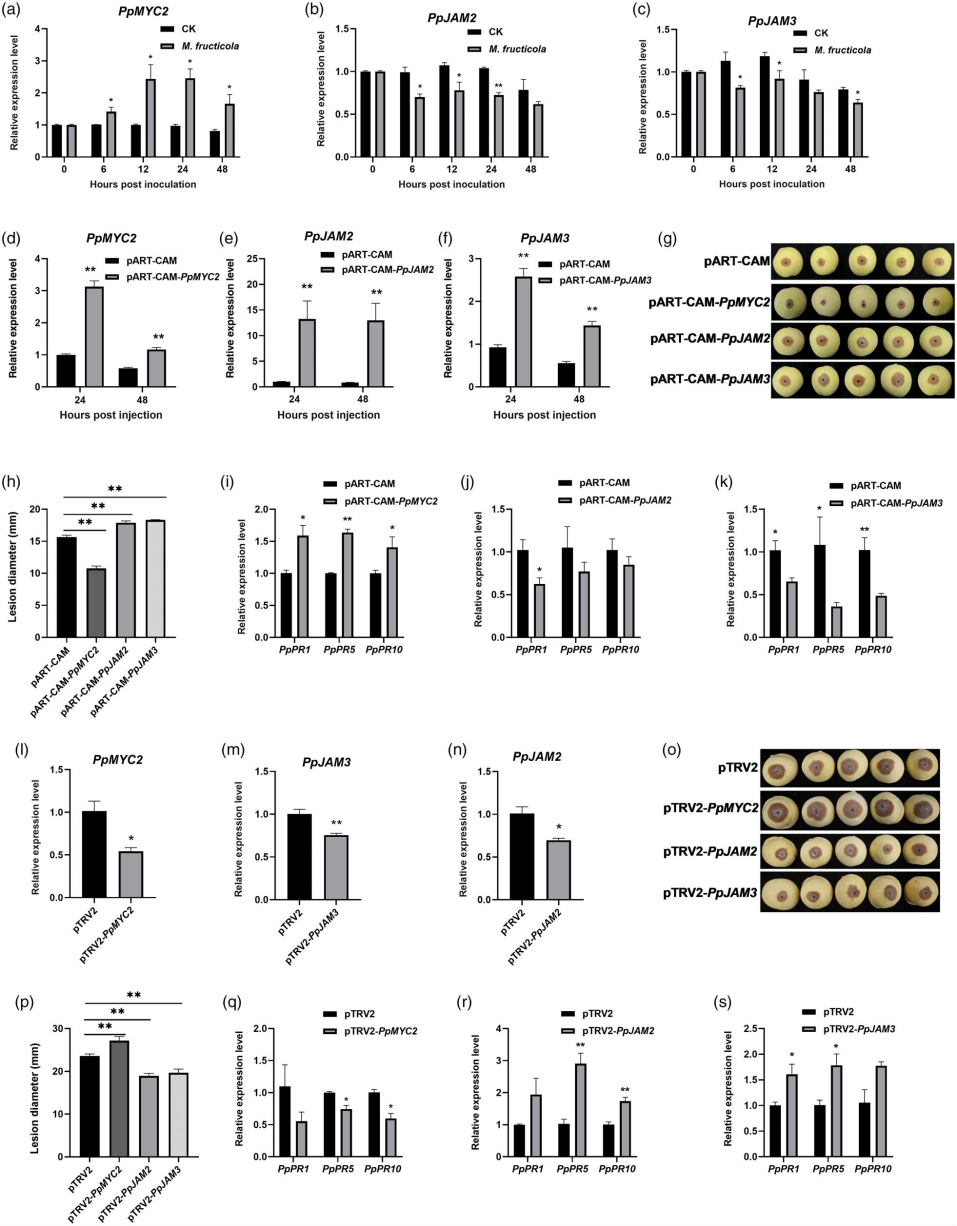
Disease resistance of peach fruit after transient overexpression and silencing of *PpMYC2* and *PpJAM2/3*. (a‐c) Expression patterns of *PpMYC2* and *PpJAM2/3* at different time points in peach fruit after inoculation with sterile water (CK) or *M. fructicola*. Data are presented as mean ± SE (*n* = 3). (d‐f) The transcription levels of *PpMYC2* and *PpJAM2/3* in the overexpression peach fruit. The empty pART‐CAM vector was used as the control. Data are presented as mean ± SE (*n* = 6). (g‐h) The phenotype and lesion diameter of the overexpression peach fruit at 72 h after *M. fructicola* inoculation. Peach fruit was inoculated with *M. fructicola* at 48 h after transient transformation with overexpression vectors. Data are presented as mean ± SE (*n* = 3). (i‐k) The transcription levels of pathogenesis‐related protein (PR) genes in peach fruit following transient overexpression of *PpMYC2* and *PpJAM2/3*. Data are presented as mean ± SE (*n* = 6). (l‐n) The transcription levels of *PpMYC2* and *PpJAM2/3* in the silencing peach fruit. The empty pTRV2 vector was used as the control. Data are presented as mean ± SE (*n* = 6). (o, p) The phenotype and lesion diameter of the silencing peach fruit at 72 h after *M. fructicola* inoculation. Peach fruit was inoculated with *M. fructicola* at 10 days after transient transformation with silencing vectors. Data are presented as mean ± SE (*n* = 3). (q‐s) The transcription levels of PR genes in peach fruit following transient silencing of *PpMYC2* and *PpJAM2/3*. Data are presented as mean ± SE (*n* = 6). Asterisks indicate significant differences (**, *P* < 0.01; *, *P* < 0.05; Student's *t*‐test).

To investigate whether PpMYC2 and PpJAM2/3 are involved in the response to *M. fructicola* infection, we checked their gene expression levels in peach fruit inoculated with *M. fructicola*. As shown in Figure 1a, compared with the control, the expression of *PpMYC2* increased gradually in the early stages of *M. fructicola* infection, maintained a high level at 12 and 24 h, and then decreased from 24 to 48 h. The expressions of *PpJAM2/3* began to show a significant decline at 6 h after inoculation with *M. fructicola*, and remained at a low level during the subsequent storage time (Figure 1b,c). These findings suggested that the expressions of *PpMYC2* and *PpJAM2/3* exhibited a different response to the infection of *M. fructicola* in peach fruit.

### PpMYC2 positively regulates the disease resistance of peach fruit, whereas PpJAM2/3 negatively regulates it

To explore the possible role of PpMYC2 and PpJAM2/3 in defence against *M. fructicola*, transient overexpressing and virus‐induced gene silencing (VIGS) assays in peach fruit were conducted. The expression levels of *PpMYC2 and PpJAM2/3* in peach fruit transformed with recombinant overexpression vectors exhibited significant increases compared with the control (pART‐CAM), indicating their successful overexpression in peach fruit (Figure 1d–f). Additionally, the expression levels of *PpMYC2 and PpJAM2/3* in peach fruit transformed with recombinant silencing vectors significantly decreased compared with the control (pTRV2), confirming their effective silencing in peach fruit (Figure 1l–n). As shown in Figure 1g,h, transient overexpression of *PpMYC2* delayed the disease progression and significantly decreased the lesion diameter of peach fruit, whereas transient overexpression of *PpJAM2*/*3* accelerated the disease progression and increased lesion diameter. In contrast, silencing *PpMYC2* transiently sped up disease expansion, whereas silencing *PpJAM2/3* transiently slowed it down notably (Figure 1o,p). In addition, we determined the transcriptional changes of the disease resistance marker genes, which are the PR genes *PpPR1*, *PpPR5* and *PpPR10*. The transcriptional levels of *PpPR1*, *PpPR5* and *PpPR10* were increased in peach fruit with *PpMYC2* overexpression or *PpJAM2/3* silencing (Figure 1i–k). Conversely, the silencing of *PpMYC2* or the overexpression of *PpJAM2/3* in peach fruit resulted in decreased expression levels of these three PR genes (Figure 1q–s). These results indicated that PpMYC2 positively regulated the disease resistance of peach fruit, whereas PpJAM2/3 negatively regulated it.

### Identification of the target genes of PpMYC2

**Figure 2:**
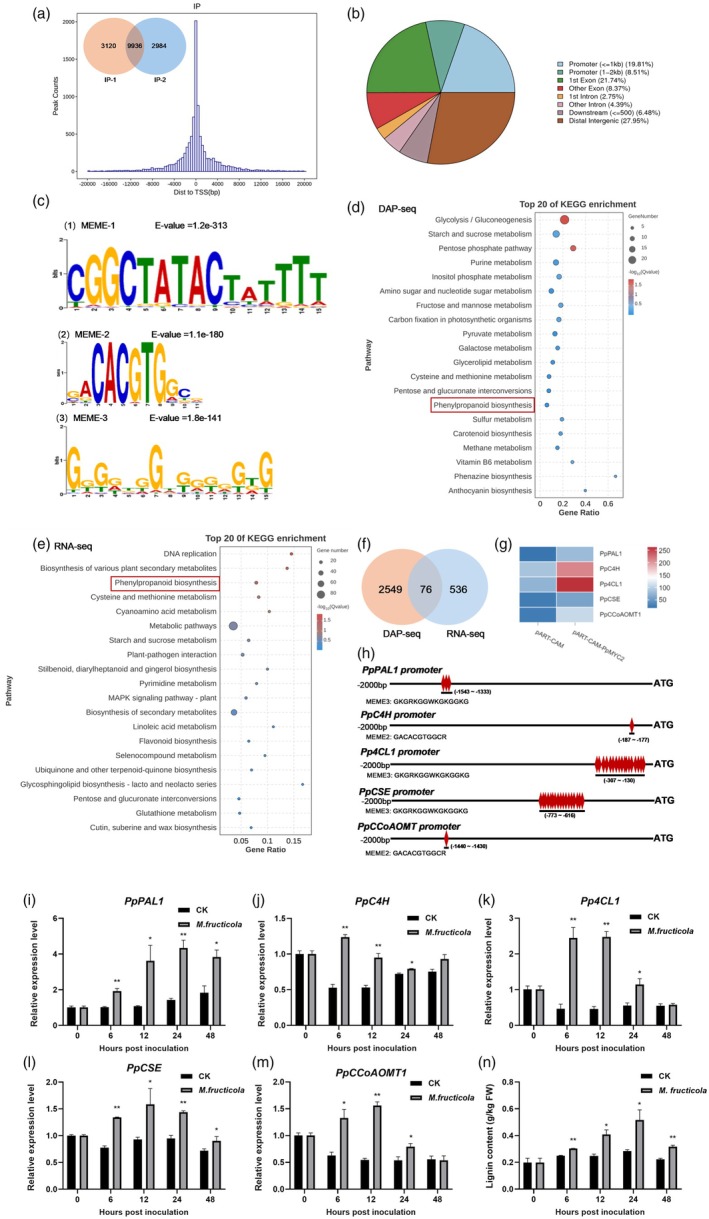
Combination analysis of DAP‐seq and RNA‐seq identified the potential target genes of PpMYC2. (a) Overlapping peaks and distance from the centre of the binding site to transcription start site (TSS) for all peaks. (b) Distribution of PpMYC2‐binding peaks on gene functional elements. (c) The top three conserved motifs enriched within the recognition interval of PpMYC2. (d) Kyoto Encyclopedia of Genes and Genomes (KEGG) pathway enrichment analysis of PpMYC2‐binding genes. The gene ratio represents the number of peak‐associated genes in each pathway as a percentage of the number of all peak‐associated genes. (e) KEGG pathway enrichment analysis of differentially expressed genes (DEGs) between the control and *PpMYC2*‐overexpressed peach fruit. The gene ratio represents the number of DEGs in each pathway as a percentage of the number of all DEGs. (f) Venn diagram of intersection DEGs by DAP‐seq and RNA‐seq. The genes on promoters bound by PpMYC2 were used for intersection analysis with the DEGs of RNA‐seq. (g) The heatmap of DEGs (*PpPAL1*, *PpC4H*, *Pp4CL1*, *PpCSE* and *PpCCoAOMT1*) between the control and *PpMYC2*‐overexpressed peach fruit. The empty pART‐CAM vector was used as the control. Blue indicates low expression and red indicates high expression. (h) The motifs in the promoters of *PpPAL1*, *PpC4H*, *Pp4CL1*, *PpCSE* and *PpCCoAOMT1*. The promoter length and the motifs are indicated with black lines and red diamonds, respectively. (i‐n) Expression patterns of *PpPAL1*, *PpC4H*, *Pp4CL1*, *PpCSE* and *PpCCoAOMT1* and lignin content at different time points in peach fruit inoculated with sterile water or *M. fructicola*. Data are presented as mean ± SE (*n* = 3). Asterisks indicate significant differences (**, *P* < 0.01; *, *P* < 0.05; Student's *t*‐test).

To investigate the potential target genes of PpMYC2, we conducted a comprehensive DNA affinity purification sequencing (DAP‐seq) analysis at the genomic level. Two DAP‐seq replicates identified 9936 overlapping peaks, which we considered as high‐confidence binding regions for subsequent analysis (Figure 2a, Table S1). By calculating the frequency distribution of the PpMYC2 binding region, we found that PpMYC2 tended to bind to DNA sequences near the transcription start site (TSS). By analysing the peak distribution, we discovered that 28.32% of these peaks were located in the 0 ~ 2000 bp promoter region upstream of the TSS (Figure 2b), whose annotated genes were used for subsequent screening of PpMYC2 target genes. Furthermore, we predicted the recognition motifs of PpMYC2 using MEME Suite (Table S2). Figure 2c revealed the top three significantly enriched motifs. Among them, MEME‐3 (5'‐GKGRKGGWKGKGGKG‐3′) showed the highest binding frequency, accounting for 19.87% of the total, and MEME‐2 contained the typical core‐binding element (5'‐CACGTG‐3′) or its variant (5’‐CACATG‐3′). Kyoto Encyclopedia of Genes and Genomes (KEGG) enrichment analysis of peak‐related genes showed that primary metabolic processes, such as starch and sugar metabolism, and glycolysis/gluconeogenesis, as well as secondary metabolic processes, including phenylpropanoid biosynthesis, ascorbic acid and aldarate metabolism, and carotenoid synthesis were significantly enriched (Figure 2d).

To further clarify PpMYC2‐regulated target genes, RNA‐seq analysis was performed with *PpMYC2*‐overexpressed peach fruit and the control. A total of 612 differentially expressed genes (DEGs) were identified, including 266 up‐regulated and 346 down‐regulated DEGs (Figure S4, Table S3). KEGG enrichment analysis showed the 612 DEGs were involved in disease resistance pathways, including biosynthesis of secondary metabolites, phenylpropanoid biosynthesis, MAPK signalling, flavonoid biosynthesis and plant‐pathogen interaction (Figure 2e). It is worth noting that phenylpropanoid biosynthesis was enriched in both DAP‐seq and RNA‐seq. Further analysis revealed that there were 75 overlap genes between the genes in the promoters bound by PpMYC2 from DAP‐seq and the DEGs from RNA‐seq (Figure 2f, Table S4). Among 75 overlap genes, *PpPAL1*, *PpC4H*, *Pp4CL1*, *PpCSE* and *PpCCoAOMT1* in the phenylpropanoid biosynthesis pathway, were significantly up‐regulated in *PpMYC2*‐overexpressed peach fruit (Figure 2g). These five genes play crucial roles in lignin synthesis through the phenylpropanoid biosynthesis pathway. Figure 2h showed the motif position of the promoters of these five genes. The binding motifs of *PpPAL1*, *Pp4CL1* and *PpCSE* were identified as MEME‐3, and the binding motifs of *PpC4H* and *PpCCoAOMT1* were identified as MEME‐2. Furthermore, we monitored the changes in expression levels of these five genes and lignin content in peach fruit after infection with *M. fructicola*. Results showed that *M. fructicola* induced the up‐regulation of *PpPAL1*, *PpC4H*, *Pp4CL1*, *PpCSE* and *PpCCoAOMT1*, accompanied by increased lignin content (Figure 2i–n). Notably, at 6, 12 and 24 h after infection, their expression levels and lignin accumulation were sustained at high levels, which were similar to *PpMYC2* expression and contrary to the expressions of *PpJAM2/3*. PpJAM2/3 and PpMYC2 all belong to the family of bHLH transcription factors containing the conserved HLH domain, suggesting that they may share a common binding region of the target genes. Therefore, these five genes were further analysed as potential target genes of PpMYC2 and PpJAM2/3.

### PpMYC2 and PpJAM2/3 regulate lignin synthesis by influencing the expressions of lignin synthesis genes

**Figure 3:**
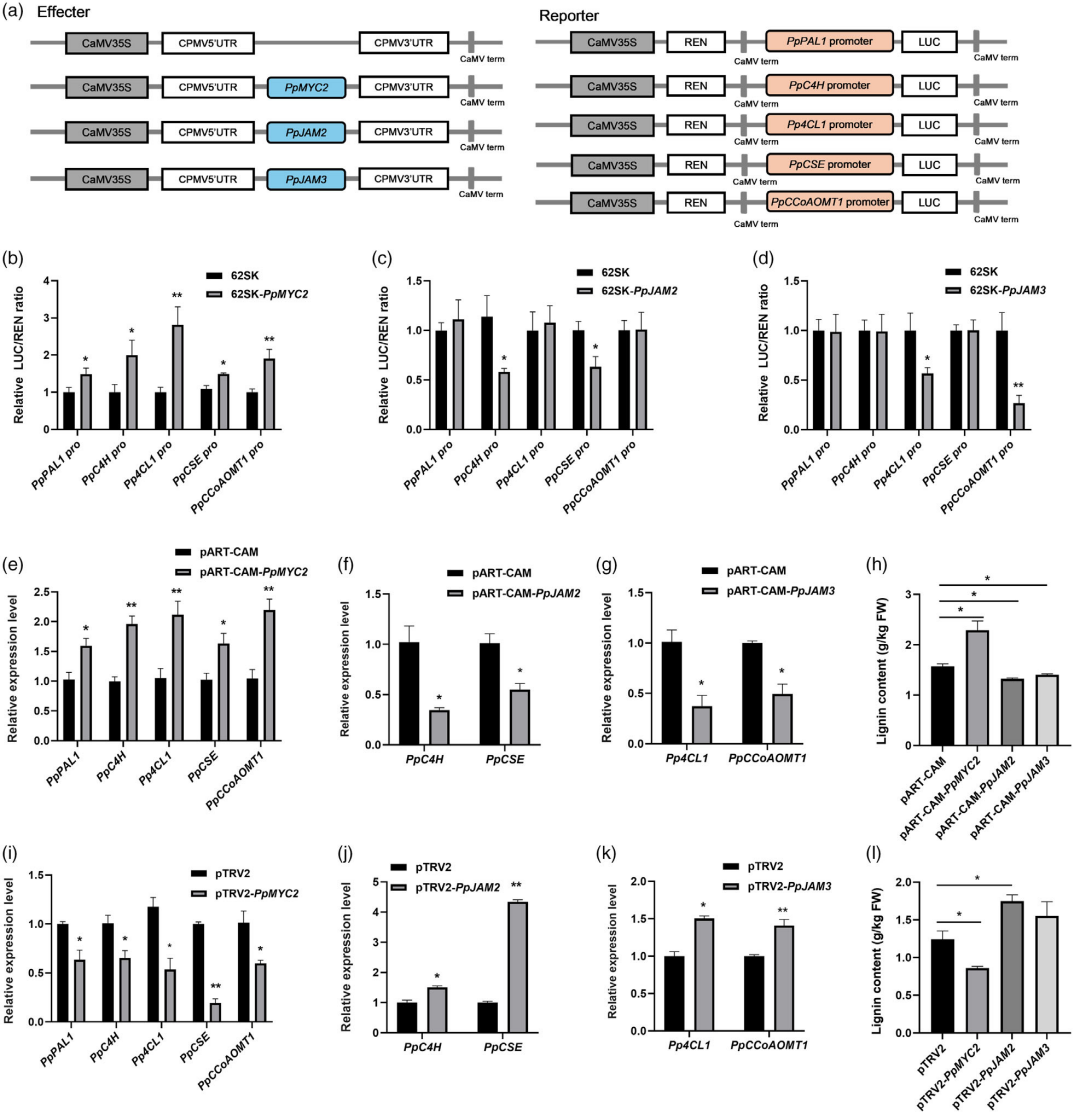
Effects of PpMYC2 and PpJAM2/3 on the transcription activities of lignin synthesis genes. (a) Schematic diagram of the effector and reporter constructs for dual‐luciferase reporter (DLR) assay. (b–d) Effects of PpMYC2 and PpJAM2/3 on transcriptional activities of lignin synthesis genes in tobacco leaves. The ratio of LUC/REN of the empty vector (62SK) plus promoter was used as a calibrator (set as 1). Data are presented as mean ± SE (*n* = 6). (e–h) The transcription levels of lignin synthesis genes and lignin content in peach fruit with transient overexpression of *PpMYC2 and PpJAM2/3*. The data are expressed as mean ± SE (*n* = 6). (i–l) The expression levels of lignin synthesis genes and lignin content in peach fruit with transient silencing of *PpMYC2 and PpJAM2/3*. Data are expressed as mean ± SE (*n* = 6). Asterisks indicate significant differences (**, *P* < 0.01; *, *P* < 0.05; Student's *t*‐test).

Dual‐luciferase reporter (DLR) assay was performed to explore the impact of PpMYC2 and PpJAM2/3 on the transcription of *PpPAL1, PpC4H, Pp4CL1*, *PpCSE* and *PpCCoAOMT1*. The construction of the effector and reporter for DLR assay was shown in Figure 3a. The relative LUC/REN values were significantly higher in the presence of PpMYC2 than in the control (62SK), indicating that PpMYC2 could activate the transcription of *PpPAL1*, *PpC4H*, *Pp4CL1*, *PpCSE* and *PpCCoAOMT1* (Figure 3b). Furthermore, we analysed the regulatory effects of PpJAM2/3 on the transcriptions of these five target genes. As shown in Figure 3c, PpJAM2 suppressed the transcription of *PpC4H* and *PpCSE* but had no impact on the transcription of *PpPAL1*, *Pp4CL1* and *PpCCoAOMT1*. Similarly, PpJAM3 suppressed the transcription of *Pp4CL1* and *PpCCoAOMT1*, but did not affect the transcriptions of *PpPAL1*, *PpC4H* and *PpCSE* (Figure 3d). Therefore, *PpC4H* and *PpCSE*, as target genes of PpJAM2, as well as *Pp4CL1* and *PpCCoAOMT1*, as target genes of PpJAM3, were further studied.

By transient overexpression and silencing of *PpMYC2* and *PpJAM2/3* in peach fruit, we studied the effects of these three transcription factors on the expression of their target genes. Transient overexpression of *PpMYC2* in peach fruit increased the expression levels of *PpPAL1*, *PpC4H*, *Pp4CL1*, *PpCSE* and *PpCCoAOMT1*, promoting lignin accumulation (Figure 3e,h), whereas transient overexpression of *PpJAM2* decreased the expression of *PpC4H* and *PpCSE*, resulting in a decrease in lignin content (Figure 3f,h), and transient overexpression of *PpJAM3* down‐regulated the expression of *Pp4CL1* and *PpCCoAOMT1*, reducing lignin content (Figure 3g,h). Conversely, *PpMYC2*‐silencing reduced the expression of *PpPAL1*, *PpC4H*, *Pp4CL1*, *PpCSE* and *PpCCoAOMT1*, causing a decrease in lignin content (Figure 3i,l), whereas *PpJAM2*‐silencing and *PpJAM3‐*silencing increased the expression of their target genes, accompanied by an increase in lignin content (Figure 3j–l). These results indicated that PpMYC2 positively regulated lignin synthesis by activating transcription of *PpPAL1*, *PpC4H*, *Pp4CL1*, *PpCSE* and *PpCCoAOMT1*, while PpJAM2 negatively regulated lignin synthesis by inhibiting transcription of *PpC4H* and *PpCSE*, and PpJAM3 exerted a negative regulatory effect on lignin synthesis by suppressing the transcription of *Pp4CL1* and *PpCCoAOMT1*.

### PpMYC2 and PpJAM2/3 directly bind to the promoters of their target genes

**Figure 4:**
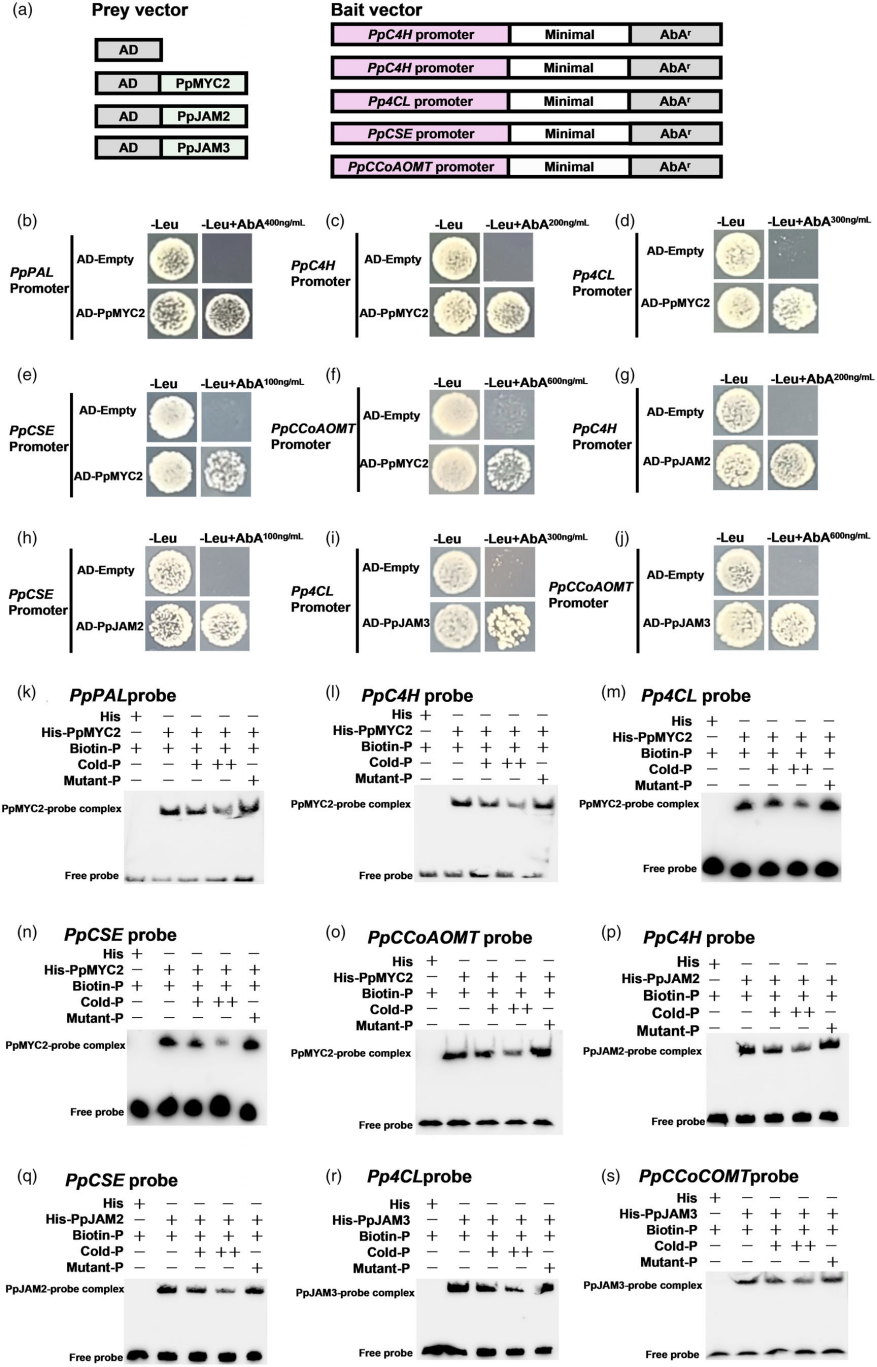
The interaction of PpMYC2 and PpJAM2/3 with the promoters of the target genes in *vitro* and *vivo*. (a) Schematic diagram of the bait and the prey constructs for yeast one‐hybrid (Y1H) assay. (b‐j) Y1H assay showing the binding of PpMYC2 and PpJAM2/3 to the promoters of the target genes. AD‐Empty was used as the negative control. (k‐s) Electrophoretic mobility shift assay (EMSA) showing the binding of PpMYC2 and PpJAM2/3 to the promoters of the target genes. The unlabelled probes served as competitors. The symbols ‘‐’, ‘+’ and ‘+ +’ indicate the absence, presence and increasing amounts, respectively.

To further confirm the direct binding of PpMYC2 and PpJAM2/3 to the promoters of their target genes, we perform a yeast one‐hybrid (Y1H) assay and an electrophoretic mobility shift assay (EMSA). The construction of the prey vector and bait vector for Y1H is shown in Figure 4a. The results revealed that the negative control and yeast cells transformed with *PpMYC2* and *PpJAM2/3* exhibited survival on SD/−Leu (Figure 4b–j). Nevertheless, only yeast cells transformed with *PpMYC2* and *PpJAM2/3* were able to survive when cultured on SD/−Leu containing Aureobasidin A (AbA). This observation demonstrated the interaction between these three transcription factors and the promoter sequences of their respective target genes.

The probes of *PpPAL1*, *PpC4H*, *Pp4CL1*, *PpCSE* and *PpCCoAOMT1* were designed based on the predicted binding motif with the highest score value derived from DAP‐seq data (Table S5). As expected, when the His‐PpMYC2 recombinant protein was co‐incubated with the biotin‐labelled probes of *PpPAL1*, *PpC4H*, *Pp4CL1*, *PpCSE* and *PpCCoAOMT1*, a lagging band appeared, but His protein did not (Figure 4k–o). Similarly, a lagging band was observed when His‐PpJAM2 recombinant protein was co‐incubated with biotin‐labelled probes of *PpC4H* and *PpCSE* (Figure 4p,q), and when His‐PpJAM3 was co‐incubated with biotin‐labelled probes of *Pp4CL1* and *PpCCoAOMT1* (Figure 4r–s). Moreover, with the increase in the amounts of cold probes, the intensity of the mobility shift signals diminished. The addition of the mutated probes did not affect the binding of these three recombinant proteins and the biotin‐labelled probes. In conclusion, the above results demonstrated that PpMYC2 and PpJAM2/3 can bind to the specific cis‐elements in the promoters of their respective target genes, and there exists a consensus binding site present in the promoters of their common target genes.

### PpMYC2 and PpJAM2/3 participate in the disease resistance of Arabidopsis by regulating lignin synthesis

**Figure 5:**
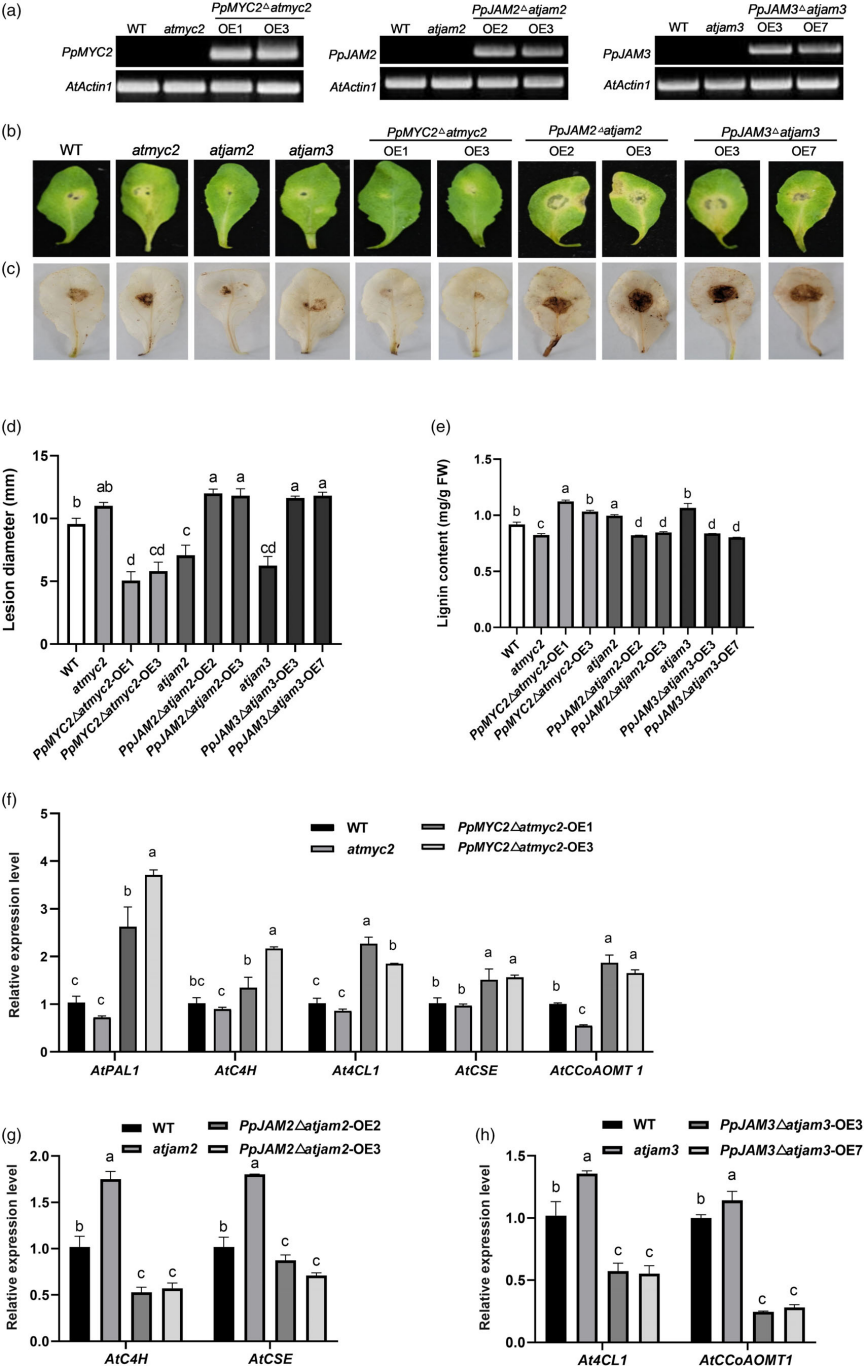
Disease resistance of Arabidopsis from mutants, wild type (WT) and overexpression lines. (a) Identification of transgenic Arabidopsis. (b) The phenotype of Arabidopsis leaves from different lines at 2 days after infection with *M. fructicola*. (c) DAB staining of Arabidopsis leaves from different lines at 2 days after infection with *M. fructicola*. (d) Lesion diameter of Arabidopsis leaves from different lines at 2 days after infection with *M. fructicola*. (e) Lignin content in mutants, WT and overexpression lines. The leaves of 4‐week‐old Arabidopsis were used to determine lignin content. (f‐h) Expression levels of lignin synthesis genes in mutants, WT and overexpression lines. The leaves of 4‐week‐old Arabidopsis were used to determine lignin content. Data are presented as mean ± SE (*n* = 3). Distinct letters are assigned to show significant differences (*P* < 0.05; Tukey's HSD test).

To further confirm the role of PpMYC2 and PpJAM2/3 in disease resistance, we obtained the Arabidopsis homologous gene mutants *atmyc2*, *atjam2* and *atjam3*. We overexpressed *PpMYC2*, *PpJAM2* and *PpJAM3* to Arabidopsis *atmyc2*, *atjam2* and *atjam3*, respectively. After screening, we obtained homozygous transgenic plants and subsequently identified positive transgenic lines using semi‐quantitative RT‐PCR. As shown in Figure 5a, the amplified target genes were observed only in transgenic lines, but not in wild type (WT) and mutants, indicating that the positive lines were successfully obtained. Arabidopsis leaves from different lines were inoculated with *M. fructicola* at 4 weeks old. Compared with WT, the *atmyc2* mutant exhibited more severe disease symptoms and a larger rot diameter (Figure 5b,d). Conversely, *atjam2* and *atjam3* mutants displayed mitigated disease symptoms and a smaller rot diameter. In agreement with the disease phenotype, 3,3′‐diaminobenzidine (DAB) staining revealed that the *atmyc2* mutant accumulated higher H_2_O_2_ at the lesion site, whereas the *atjam2* and *atjam3* mutants displayed a reduction in H_2_O_2_ accumulation (Figure 5c). By overexpressing *AtMYC2* in the *atmyc2* mutant, disease symptoms were significantly alleviated, as well as a reduction in rot diameter and H_2_O_2_ accumulation, whereas overexpressing *PpJAM2* in the *atjam2* mutant or overexpressing *PpJAM3* in the *atjam3* mutant accelerated the development of disease symptoms in Arabidopsis leaves, accompanied by an increase in disease diameter and H_2_O_2_ accumulation (Figure 5b–d).

To investigate whether these three transcription factors influenced disease resistance of Arabidopsis by regulating lignin synthesis, we determined the expression levels of lignin synthesis‐related genes and lignin content in different Arabidopsis lines. Compared with WT, the *atmyc2* mutant displayed lower expression levels of *AtPAL1*, *AtC4H*, *At4CL1, AtCSE* and *AtCCoAOMT1*, along with lower lignin content (Figure 5e,f). In contrast to the *atmyc2* mutant, the *atjam2* mutant showed higher expression levels of *AtC4H* and *AtCSE*, accompanied by an increase in lignin content (Figure 5e,g), and the *atjam3* mutant exhibited higher expression levels of *At4CL1* and *AtCCoAOMT1*, along with higher lignin accumulation (Figure 5e,h). By complementing *PpMYC2* in the *atmyc2* mutant, we observed increased expression levels of *AtPAL1*, *AtC4H*, *At4CL1*, *AtCSE* and *AtCCoAOMT1* and lignin content (Figure 5e,f). In contrast, compared with the *atjam2* mutant, a reduction in lignin content and the expression levels of *AtC4H* and *AtCSE* were detected in *PpJAM2*∆*atjam2*‐OE lines (Figure 5g). Similarly, *PpJAM3*∆*atjam3*‐OE lines showed decreased expression levels of *At4CL* and *AtCCoAOMT1* and lignin content compared with the *atjam3* mutant (Figure 5e,h). In a word, PpMYC2 positively regulated the resistance of Arabidopsis to *M. fructicola* by promoting lignin synthesis, whereas PpJAM2/3 exhibited negative regulatory effects by suppressing lignin synthesis.

### PpJAM2/3 interfered with the activation of PpMYC2 on their common target genes by competing for the same binding sites

**Figure 6:**
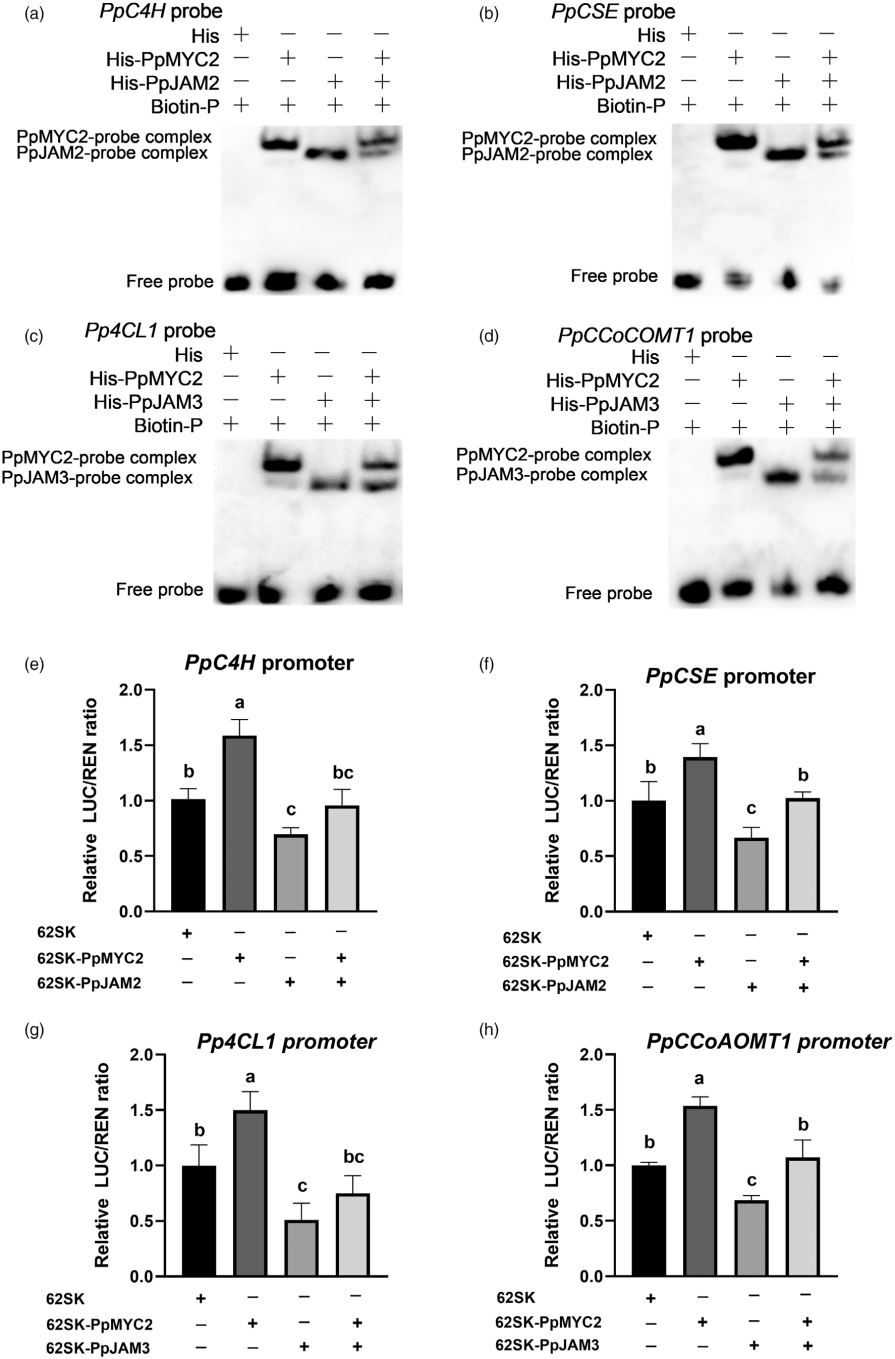
PpJAM2/3 interfere with the transcription of target genes by competitively binding to the promoters with PpMYC2. (a‐d) Electrophoretic mobility shift assay (EMSA) showing the competitive binding of PpJAM2/3 and PpMYC2 to the promoters of common target genes. His protein served as a negative control. (e‐h) PpJAM2/3 interfered with PpMYC2 in activating the transcription of common target genes. The symbols ‘‐’ and ‘+’ indicate the absence and presence, respectively. Data are presented as mean ± SE (*n* = 6). Distinct letters are assigned to show significant differences (*P* < 0.05; Tukey's HSD test).

Since PpJAM2/3 could bind to the same promoter sequence with PpMYC2, we speculated that PpJAM2/3 may affect PpMYC2 binding to the promoter, so we did a competitive EMSA to test this. As shown in Figure 6a–d, with the addition of His‐PpJAM2 or His‐PpJAM3, the binding interaction between His‐PpMYC2 and the labelled probe was diminished, and a binding band of His‐PpJAM2 or His‐PpJAM3 and the labelled probe appeared, indicating that His‐PpJAM2 or His‐PpJAM3 competed with His‐PpMYC2 to bind the same promoter sequence. PpJAM2/3 regulated target genes in opposite ways to PpMYC2 by competitively binding their promoters. We speculated that PpJAM2/3 might influence the activation of PpMYC2 on their common target genes. We used a DLR assay to verify this. The result revealed that PpJAM2 prevented PpMYC2 from activating the transcription of *PpC4H* and *PpCSE* (Figure 6e,f). Similarly, PpJAM3 prevented PpMYC2 from activating the transcription of *Pp4CL1* and *PpCCoAOMT1* (Figure 6g,h). These results highlighted an antagonistic relationship between PpMYC2 and PpJAM2/3 in regulating the expression of lignin synthesis genes.

## Discussion

### The different functions of PpMYC2 and PpJAM2/3 in regulating disease resistance of peach fruit

It has been reported that the MYC2 transcription factor participates in a range of stress responses, including chilling, salt, drought and biotic stress, but previous studies focused predominantly on a few model crops, such as Arabidopsis, rice and tomatoes (Hu *et al*., 2024; Zhao *et al*., 2013). In this study, we identified and characterized PpMYC2 and PpJAM2/3 in peaches. The expression pattern analysis revealed that *M. fructicola* infection up‐regulated *PpMYC2* expression, but down‐regulated the expression of *PpJAM2/3* (Figure 1a–c). Interestingly, our results are partially similar to those of *MYC2* and *JAMs* observed in other plants. In Arabidopsis, the expression of *AtMYC2* and *AtJAM1/2* was induced by pathogen, but the expression of *AtJAM3* was constitutive (Sasaki‐Sekimoto *et al*., 2013). However, in tomato, the expression of *SlMYC2* and *SlMTB1/2/3* was all induced by *B. cinerea* (Liu *et al*., 2019). Zou *et al*. (2023) reported that *Meloidogyne incognita* induced *SlJAM1/2/3* expression, but their protein levels were significantly reduced. Although homologous genes often have similar expression patterns in different species, differentiation has also been found in different species. For example, *AtPRX9* and *AtPRX40* exhibited specific expression in the tapetum of Arabidopsis, whereas *GhPRX9 and GhPRX40* in cotton were specifically expressed in the middle layer (Jacobowitz *et al*., 2019; Li *et al*., 2024). Additionally, the expression differentiation of homologous genes may be attributed to different pathogen species. For example, gene expression of a MYB‐like HTH transcription factor in *citrus* after infection with *CTV‐B2* exhibited significant down‐regulation, whereas it was up‐regulated in response to *CaLas‐B232* infection (Fu *et al*., 2016).

Studies have shown that overexpression or silencing *of MYC2* in plants has a significant impact on their disease resistance. In Arabidopsis, *myc2* mutant lines exhibited resistance exclusively to virulent pathogens, whereas plants overexpressing *AtMYC2* displayed heightened resistance to a wide range of both virulent and avirulent bacterial pathogen strains, suggesting that MYC2 has a dual role in regulating disease responses (Gautam *et al*., 2021). The knockout of *SlMYC2* in tomato fruit or *SlMYC2* RNAi of tomato plants led to susceptibility to *B. cinerea* (Du *et al*., 2017; Min *et al*., 2020). Similarly, Xiao *et al*. (2024) reported that the knockout of *NtMYC2a* in tobacco led to decreased resistance against *Ralstonia solanacearum*. Uji *et al*. (2016) found that rice overexpressing *OsMYC2* exhibited significantly enhanced defence capability against the bacterial pathogen *Xanthomonas oryzae pv. oryzae*. In our study, Arabidopsis *atmyc2* mutant and peach fruit silencing *PpMYC2* showed reduced defence against *M. fructicola*. On the contrary, overexpressing *PpMYC2* in peach fruit and *atmyc2* mutant lines enhanced disease resistance (Figures 1g,k and 6b), suggesting that PpMYC2 positively regulated the resistance to *M. fructicola*. In contrast to PpMYC2, PpJAM2/3 negatively regulated disease resistance to *M. fructicola* (Figures 1o,p and 6b). Similarly, in tomato plants, SlMTBs negatively regulated the resistance to *B. cinerea* and *M. incognita* (Liu *et al*., 2019). In Arabidopsis, *AtJAM2* overexpression attenuated the resistance to *S. exiga* and *B. cinerea* (Huang *et al*., 2018; Zou *et al*., 2023). Although PpJAM2/3 play a negative role in peach fruit disease resistance, their gene transcription levels were down‐regulated after *M. fructicola* infection. This could suggest that peach fruit employs a self‐protection or adaptive strategy in response to biotic stress.

### PpMYC2 and PpJAM2/3 participated in disease response by regulating lignin synthesis

DAP‐seq is a high‐throughput method for identifying transcription factor binding sites across the genome, characterized by its rapidity, cost‐effectiveness and independence from antibody dependence (O'Malley *et al*., 2016). Using DAP‐seq technology for genome‐wide scanning, we identified the recognition sites of PpMYC2. Interestingly, in addition to the G‐box (5’‐CACGTG‐3′) motif and G‐box variant (5’‐CACATG‐3′) that have been reported (Kazan and Manners, 2013), we also found many other unreported motifs, such as the MEME‐3 motif (5’‐GKGRKGGWKGKGGKG‐3′) that accounted for a large proportion of binding sites (Figure 2c).

A combined analysis of DAP‐seq and RNA‐seq identified five target genes (*PpPAL1, PpC4H, Pp4CL1, PpCSE* and *PpCCoAOMT1*) of PpMYC2 (Figure 2g). These five genes have been reported to participate in peach fruit lignin synthesis. Ji *et al*. (2021) reported that PpWRKY70 activated the expression of *PpPAL1* and *Pp4CL1*, resulting in lignin accumulation in peach fruit. Peach varieties with higher lignin content were also accompanied by higher *Pp4CL1* expression (Wang *et al*., 2018). PpMYB306 repressed the expression of *PpC4H*, accompanied by a lower lignin content (Li *et al*., 2023b). The expression level of *PpCSE* exhibited a strong correlation with the degree of lignification in the endocarp during the development of peach fruit (Liu *et al*., 2017). CCoAOMT, a kind of methyltransferase, is crucial for lignin biosynthesis in peaches (Li *et al*., 2021). During *M. fructicola* infection, we observed an increase in the expression levels of *PpPAL1*, *PpC4H*, *Pp4CL1*, *PpCSE* and *PpCCoAOMT1* in peach fruit, accompanied by the accumulation of lignin (Figure 2h–m). This suggested that lignin played a crucial role in the response to *M. fructicola* infection.

Furthermore, we demonstrated that PpMYC2 activated the transcription of *PpPAL1, PpC4H, Pp4CL1, PpCSE* and *PpCCoAOMT1* by binding to their promoters (Figures 3b and 4b–f,k–o). Conversely, by binding to the same promoter sequences as PpMYC2, PpJAM2 inhibited the expression of *PpC4H* and *PpCSE* (Figures 3c and 4g,h,p,q), while PpJAM3 repressed the expression of *Pp4CL1* and *PpCCoAOMT1* (Figures 3d and 4i,j,r,s). These results suggested that PpJAM2/3 partially modulated the target genes of PpMYC2. Their distinction in regulating the expression of target genes may be attributed to their differences in the acid activation domain, with the acid domains of PpJAM2/3 being less conserved compared with that of PpMYC2 (Figure S2), which is similar to PpMYC2 and PpJAM2/3 of Arabidopsis (Nakata *et al*., 2013). In Arabidopsis, AtJAM1/2/3 negatively regulated the expression of JA metabolic genes and anthocyanin‐related genes, but PpMYC2 positively regulated them (Sasaki‐Sekimoto *et al*., 2013). It has been reported that transcription factors influenced fruit's resistance to disease by modulating lignin synthesis. In peach fruit, PpMYB306 negatively regulated the resistance against *Rhizopus stolonifer* by suppressing the expression of key genes related to lignin biosynthesis (*PpC4H*, *PpCAD1* and *PpPOD44*), along with a reduction in lignin content (Li *et al*., 2023b). CsERF1B bolstered citrus fruit's resistance to *B. cinerea* by up‐regulating the transcription of lignin synthesis genes and increasing lignin accumulation (Li *et al*., 2023a). GhODO1 bound the promoter of *Gh4CL1* to enhance its expression, resulting in improved resistance against *Verticillium dahlia* (Zhu *et al*., 2022). In our study, we found that the activation of genes related to lignin synthesis by PpMYC2 promoted lignin accumulation, thereby enhancing resistance to *M. fructicola*. Conversely, the inhibition of genes related to lignin synthesis by PpJAM2/3 resulted in the decrease of lignin contents, subsequently decreasing resistance to *M. fructicola*. Collectively, PpMYC2 and PpJAM2/3 played crucial roles in lignin biosynthesis, thereby influencing the disease resistance of peach fruit.

### Antagonistic action between PpMYC2 and PpJAM2/3 is crucial for modulating the disease defence of peach fruit

**Figure 7:**
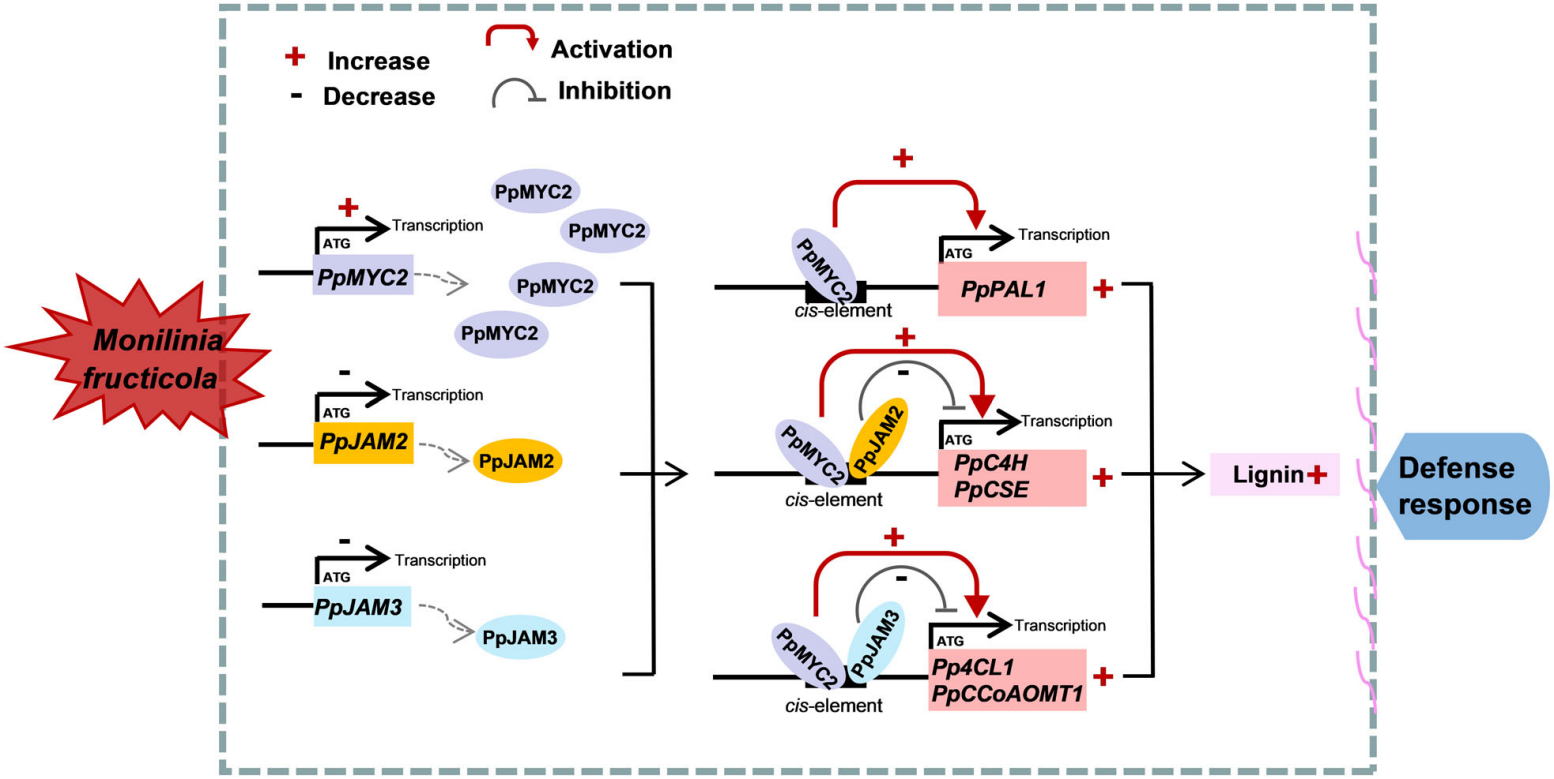
Possible molecular mechanism of PpMYC2 and PpJAM2/3 synergistically regulating disease resistance in peach fruit. The symbols ‘−’ and ‘+’ indicate the decrease and the increase, respectively. The red arrow indicates an activation effect, while the grey flat end indicates an inhibition effect. The greater the binding proportion of PpMYC2 and PpJAM2/3 to the *cis* element, the stronger the competition for binding to the promoter. After *M. fructicola* infection, the expression of *PpMYC2* in peach fruit was up‐regulated, whereas the expressions of *PpJAM2* and *PpJAM3* were down‐regulated. The up‐regulation of PpMYC2 enhanced the transcriptional activation of *PpPAL1*, *PpC4H*, *PpCSE*, *Pp4CL1* and *PpCCoAOMT1*, thereby promoting lignin accumulation. Simultaneously, the down‐regulation of *PpJAM2* decreased the transcription of *PpC4H* and *PpCSE*, and the down‐regulation of *PpJAM3* reduced the transcription of *Pp4CL1* and *PpCCoAOMT1*, collectively alleviating the inhibition of lignin synthesis. These two regulation ways ultimately resulted in an increase in lignin synthesis serving as a protective response against infection by *M. fructicola*.

Different transcription factors generally do not operate in isolation but through synergistic or antagonistic interaction (Huang *et al*., 2023). During the lignification of juice sacs in *Citrus sinensis*, *CsMYB330* expression notably was up‐regulated meanwhile *CsMYB308* expression was markedly down‐regulated. By competitively binding to the AC element in the *Cs4CL1* promoter, CsMYB330 activated the expression of *Cs4CL1*, while CsMYB308 inhibited it, thereby finely regulating juice sacs lignification (Jia *et al*., 2018). In Antirrhinum, by competitively binding to target genes, MYB305 reduced MYB340’ effective transcriptional activation, adjusting the pace of secondary metabolism in accordance with floral development and environmental changes (Moyano *et al*., 1996). In addition, BnaC09.FUL and BnaC06.WIP2 antagonistically regulated the expressions of *BnaC03.SOC1*, *BnaA08.CAL* and *BnaC03.CAL* to control the flowering time of *Brassica napus* (Min *et al*., 2025). In our study, PpJAM2/3 competed with PpMYC2 for binding to the promoters to antagonistically regulate the expressions of their common target genes. Furthermore, we demonstrated that this competitive binding interfered with PpMYC2 in activating the transcription of target genes (Figure 6a–h). Although PpJAM2/3 negatively regulated disease resistance in peach fruit, their expression levels were down‐regulated after *M. fructicola* infection. This reduction in expression attenuated their inhibition of PpMYC2‐activated transcription of lignin synthesis‐related genes, therefore reducing the inhibition of lignin synthesis. On the contrary, the expression of *PpMYC2* was up‐regulated after *M. fructicola* infection, which increased the transcription of lignin synthesis‐related genes, therefore promoting the accumulation of lignin. The two ways fine‐tuned the expression of lignin synthesis genes to promote lignin synthesis in response to *M. fructicola* infection. Figure 7 showed a working model in which PpMYC2 and PpJAM2/3 antagonistically regulated the expression of lignin synthesis genes to defend against *M. fructicola* infection. These results indicated that members of transcription factors in the bHLH family have diversified their function to adapt quickly to changes in the external environment.

## Experimental procedures

### Fruit materials and treatments

Peach fruit (*Prunus persica* L. cv. Hujingmilu) was harvested at the green and commercial maturity stages in Fenghua District, Ningbo, China. Peach fruit with uniform maturity and free from any mechanical damage was selected for the experiment.

*M. fructicola* derived from naturally infected peach fruit was inoculated onto the potato dextrose agar (PDA) medium and cultured at 25 °C for 7 days. For each peach harvested at optimum commercial maturity, two wounds (each being 3 mm wide and 3 mm deep) were made. Ten microlitres of *M. fructicola* spore suspension (1 × 10^5^ spores/mL) was inoculated into these wounds, with sterile water serving as the control. Peach fruit was stored at 20 °C under a relative humidity of 85–90%. Pulp samples within 1 cm of the inoculation site were collected at 0, 6, 12, 24 and 48 h after inoculation for subsequent analysis. Five fruits were used per replicate, with a total of three replicates.

### Bioinformatic analysis and subcellular localization of PpMYC2 and PpJAM2/3

Amino acid sequences of MYC2, JAMs and MTBs transcription factors were obtained from the following genome database: https://www.rosaceae.org/ (peach), https://solgenomics.sgn.cornell.edu/ (tomato) and https://www.arabidopsis.org/ (Arabidopsis). The phylogenetic tree was built in MEGA 7.0 by employing the neighbour‐joining method.

The CDSs (lacking a stop codon) of *PpMYC2 and PpJAM2/3* were, respectively, cloned into the pART‐CAM‐GFP vector, and then recombinant vectors were introduced into *Agrobacterium tumefaciens* strain GV3101. The transformed cells were injected into the leaves of 4‐week‐old tobacco (*Nicotiana benthamiana*). The empty pART‐CAM‐GFP vector was used as a control. After 2 days of injection, the fluorescence of tobacco leaves was observed.

### Transient overexpression and silencing of *PpMYC2* and *PpJAM2/3* in peach fruit

The full‐length CDSs of *PpMYC2* and *PpJAM2/3* were, respectively, constructed into the 35S‐driven pART‐CAM vector. The recombinant vectors were transformed into *Agrobacterium tumefaciens* strain GV3101, with the empty pART‐CAM vector serving as the control. The predicted *PpMYC2* and *PpJAM2/3* sequences with the greatest VIGS efficiency were cloned into the pTRV2 vector, respectively, and then transformed into *Agrobacterium tumefaciens* strain GV3101, with the empty pTRV2 vector serving as the control.

Mature green peach fruit was used for transient overexpression and VIGS assays. The transformed cells, after being mixed with pTRV1:pTRV2 in a 1:1 ratio, were injected into peach fruit. Each peach fruit received two injections of 1 mL per injection. All injected fruit was stored at 20 °C under a relative humidity of 85–90%. Pulp samples at the injection site were taken at 24 h and 48 h after injection for overexpression assay and at 10 days for VIGS assay, and then frozen in liquid nitrogen and stored at −80 °C for further analysis. Each peach was a replicate, with a total of six replicates.

For the remaining peach fruit in the overexpression and VIGS groups, two wounds (each being 3 mm wide and 3 mm deep) were created at the injection site. Subsequently, 10 μL of *M. fructicola* spore suspension (1 × 10^5^ spores/mL) was inoculated into these wounds at 48 h and 10 days after *Agrobacterium* GV3101 injection, respectively. At 3 days post‐inoculation, lesion diameter was measured and peach fruit were photographed. Five peaches were a replicate, with a total of three replicates.

### DAP‐seq and RNA‐seq

The full‐length CDS of *PpMYC2* was recombined into the pHalo‐tag vector for protein expression. The subsequent DAP‐seq experiments and data analysis were carried out by Genedenovo Biotechnology Co., Ltd. (Guangzhou, China), using two biological replicates. The fruit samples for RNA‐seq were collected at 24 h after the injection of the empty pART‐CAM or recombinant pART‐CAM‐*PpMYC2* vectors, with three biological replicates. DEGs were screened using a threshold of a fold change (FC) ≥ 1.5 and *P*‐value<0.05. RNA‐seq and data analysis were conducted by Genedenovo Biotechnology Co., Ltd. (Guangzhou, China).

### Y1H assay

The full‐length CDSs of *PpMYC2* and *PpJAM2/3* were, respectively, recombined into the pGADT7 vector to construct the prey vectors. The promoter sequences of *PpPAL1* (−1282 bp ~ −1632 bp), *PpC4H* (−136 bp ~ −535 bp), *PpCSE* (−576 bp ~ −825 bp), *Pp4CL1* (−68 bp ~ −332 bp) and *PpCCoAOMT1* (−1311 bp ~ −1560 bp) were, respectively, cloned into the pAbAi vector to construct the bait vectors. The bait and prey vectors were co‐transformed into Y1H Gold yeast. The pGADT7 empty vector was transformed into the bait yeast cells, which served as the negative control. AbA served as an inhibitor.

### EMSA

The CDSs (lacking a stop codon) of *PpMYC2* and *PpJAM2/3* were, respectively, cloned into pET32a to construct protein expression vectors, which were then transformed into *Escherichia coli* BL21 (DE3). The probes (shown in Table S6) were designed according to DAP‐seq and were labelled with biotin or remained unlabelled. EMSA binding analysis was performed using a Chemiluminescent EMSA Kit supplied by Beyotime biotechnology Co., Ltd.

### DLR assay

The promoter sequences of *PpPAL1*, *PpC4H*, *PpCSE*, *Pp4CL1* and *PpCCoAOMT1* were, respectively, cloned into the pGreenII 0800‐LUC vector to construct the reporters. The full‐length CDSs of *PpMYC2* and *PpJAM2/3* were, respectively, cloned into the pGreen II 62‐SK vector to construct the effectors. The empty pGreen II 62‐SK vector was used as the control effector. The *Agrobacterium tumefaciens* strain GV3101 (Psoup) transformed with the reporter and the effector was co‐infiltrated into the leaves of 4‐week‐old tobacco. After 3 days, the activities of firefly luciferase and Renilla luciferase were detected. Six biological replicates were performed.

### Construction of transgenic Arabidopsis lines and *M. fructicola* infection

All Arabidopsis lines were derivatives of the Columbia‐0 ecotype (WT). The T‐DNA lines, *atmyc2* (SALK_017005C), *atjam2* (SALK_051659C) and *atjam3* (SALK_206035C) were obtained from the AraShare Technology Service Center. After surface disinfection, Arabidopsis seeds were sown in 0.75% (w/v) agar and, pH 5.7 half Murashige and Skoog (1/2 MS) medium and grew for 7 days under a 16 h light / 8 h dark cycle at 22 °C and 65% relative humidity (RH). Subsequently, the Arabidopsis seedlings were planted in soil and cultivated as described above.

As described by the method of Clough and Bent (1998), Arabidopsis mutant *atmyc2*, *atjam2* and *atjam3* were transfected by *Agrobacterium* GV3101 containing pART‐CAM‐*PpMYC2*, pART‐CAM‐*PpJAM2* and pART‐CAM‐*PpJAM3* recombinant vectors, respectively. Homozygous transgenic Arabidopsis lines were obtained through three generations of screening. Positive lines were identified by semi‐quantitative RT‐PCR. The leaves of 4‐week‐old Arabidopsis from mutants, WT and overexpression lines were taken for determining lignin content. Remaining leaves were inoculated with 3 mm‐diameter mycelial discs. At 3 days post‐inoculation, the leaves were evaluated for disease severity and DAB staining. DAB staining was conducted by the method of Zhang *et al*. (2023).

### Determination of lignin content

Lignin content was determined using a lignin test kit (Nanjing Jiancheng Bioengineering Institute) and expressed as g·kg^−1^ fresh weight (FW).

### RNA isolation, RT‐PCR and RT‐qPCR

RNA isolation was conducted using the Eastep™ Super Total RNA Extraction Kit (Promega (Beijing) Biotech Co., Ltd). cDNA synthesis and qPCR amplifications were carried out according to the instructions of the HiScript IV RT SuperMix for qPCR Kit (+gDNA wiper) and the ChamQ Blue Universal SYBR qPCR Master Mix Kit (Nanjing Vazyme Biotech Co., Ltd). The results were normalized using the 2^−ΔΔCT^ method. *PpTEF2* and *AtActin1* were used as the internal reference genes for peach and Arabidopsis, respectively.

### Statistical analysis

Significant differences between two groups were assessed using an independent samples *t*‐test. 0.01 < *P*‐value <0.05 was indicated by a single asterisk (*), while *P*‐value <0.01 was denoted by a double asterisk (**). For comparisons involving more than two groups, Tukey's HSD test was utilised. Significant differences among the groups were represented using distinct letters (*P* < 0.05).

## Author contributions

Qian Li: Data curation; conceptualization; validation; investigation; writing—original draft. Yi Chen: Visualization; methodology; writing—review and editing. Yingying Wei: Formal analysis; supervision; funding acquisition. Shu Jiang: Visualization; formal analysis. Jianfen Ye: Formal analysis; methodology. Jiahui Chen: Data curation; validation; writing—review and editing. Feng Xu: Software; visualization; funding acquisition. Xingfeng Shao: Conceptualization; supervision; funding acquisition; project administration; writing—review and editing.

## Conflict of interest

The authors declare that they have no competing interests.

## Supporting information


**Figure S1** Phylogenetic analysis.
**Figure S2** Multiple alignments of amino acid sequences.
**Figure S3** Subcellular localization of PpMYC2 and PpJAM2/3.
**Figure S4** The DEGs statistics.

**Table S1** The list of PpMYC2‐binding peaks from two biological replicates.
**Table S2** The list of PpMYC2‐binding motifs.
**Table S3** The list of DEGs.
**Table S4** The overlapping genes between DEGs identified by RNA‐seq and PpMYC2‐target genes identified by DAP‐seq.
**Table S5** The binding motifs of target genes in DAP‐seq data.
**Table S6** The primers and probe sequences for this study.

## Data Availability

The data that support the findings of this study are available on request from the corresponding author. The data are not publicly available due to privacy or ethical restrictions.
