# Supplementary material for: PpMYC2 and PpJAM2/3 antagonistically regulate lignin synthesis to cope with the disease in peach fruit
Source: Plant Biotechnol J. 2025 Jun 4;23(9):3524–39. doi: 10.1111/pbi.70177 (PMC12392935; doi:10.1111/pbi.70177)
Supplement: Supplementary file 1 — Figure S1 Phylogenetic analysis. Figure S2 Multiple alignments of amino acid sequences. Figure S3 Subcellular localization of PpMYC2 and PpJAM2/3. Figure S4 The DEGs statistics. [file PBI-23-3524-s001.doc]

**Figure S1**


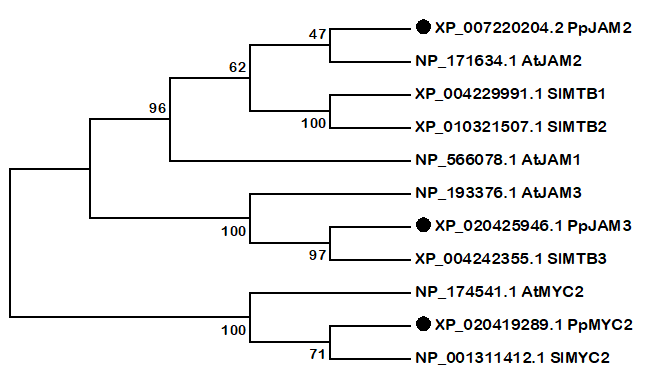


Figure S1. Phylogenetic analysis. PpMYC2, PpJAM2 and PpJAM3 are from *Prunus persica*. AtMYC2, AtJAM1, AtJAM2 and AtJAM3 are from *Arabidopsis thaliana*. SlMYC2, AtMTB1, SlMTB2, and SlMTB3 are from *Solanum lycopersicum*.

**Figure S2**

**
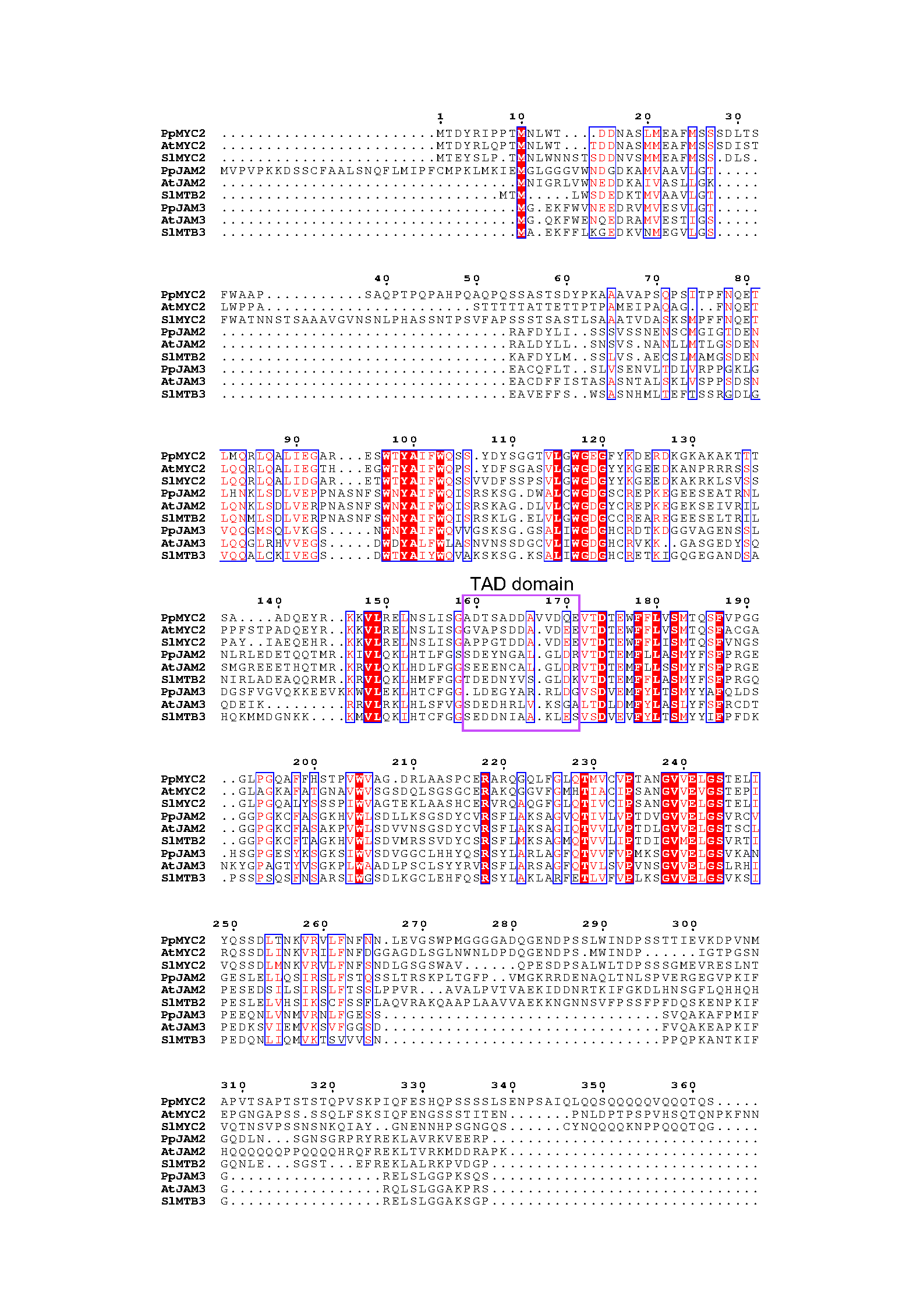
**

**
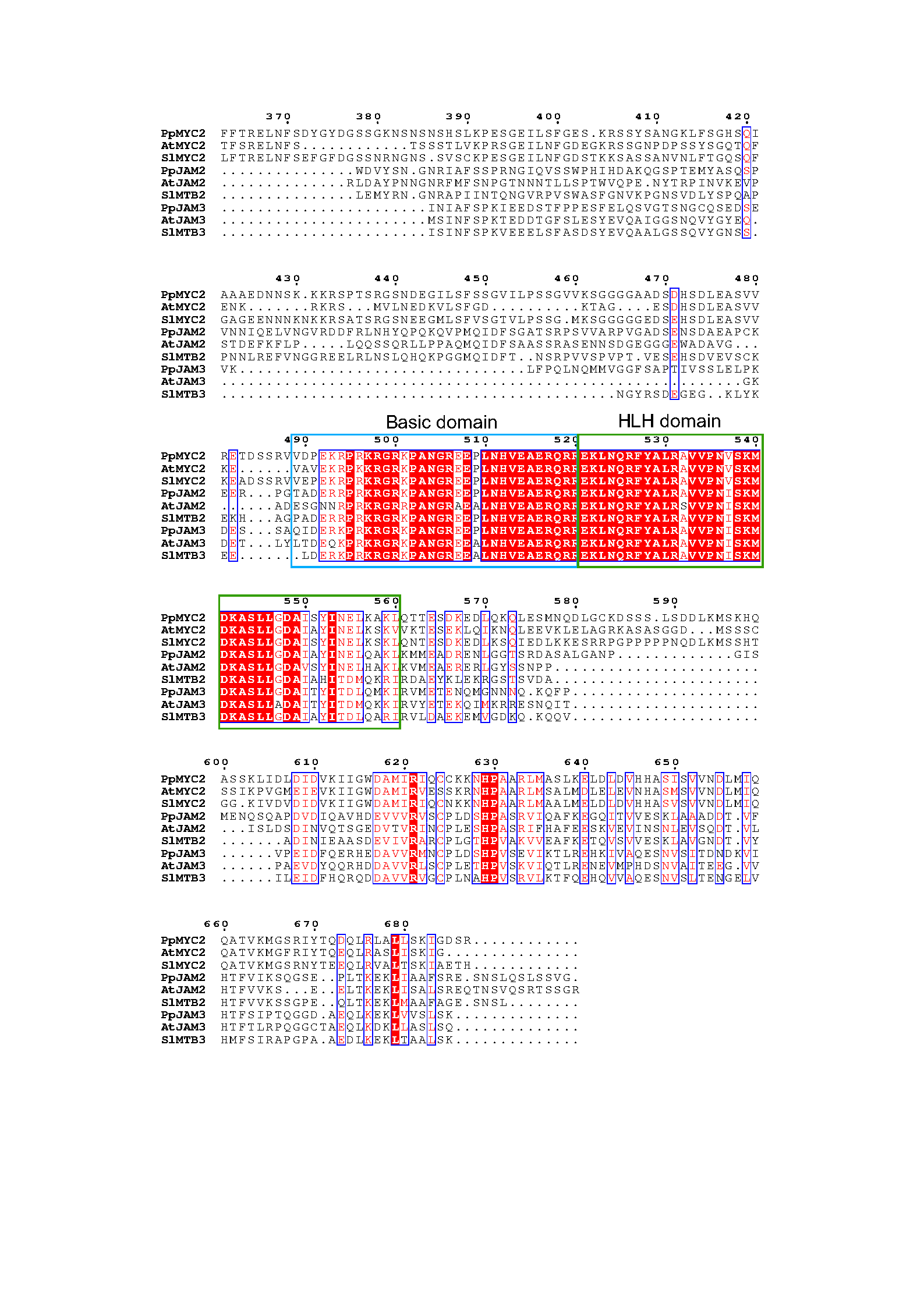
**

Figure S2. Multiple alignments of amino acid sequences of PpMYC2, PpJAM2, PpJAM3, AtMYC2, AtJAM2, AtJAM3, SlMYC2, SlMTB2, and SlMTB3.

**Figure S3**


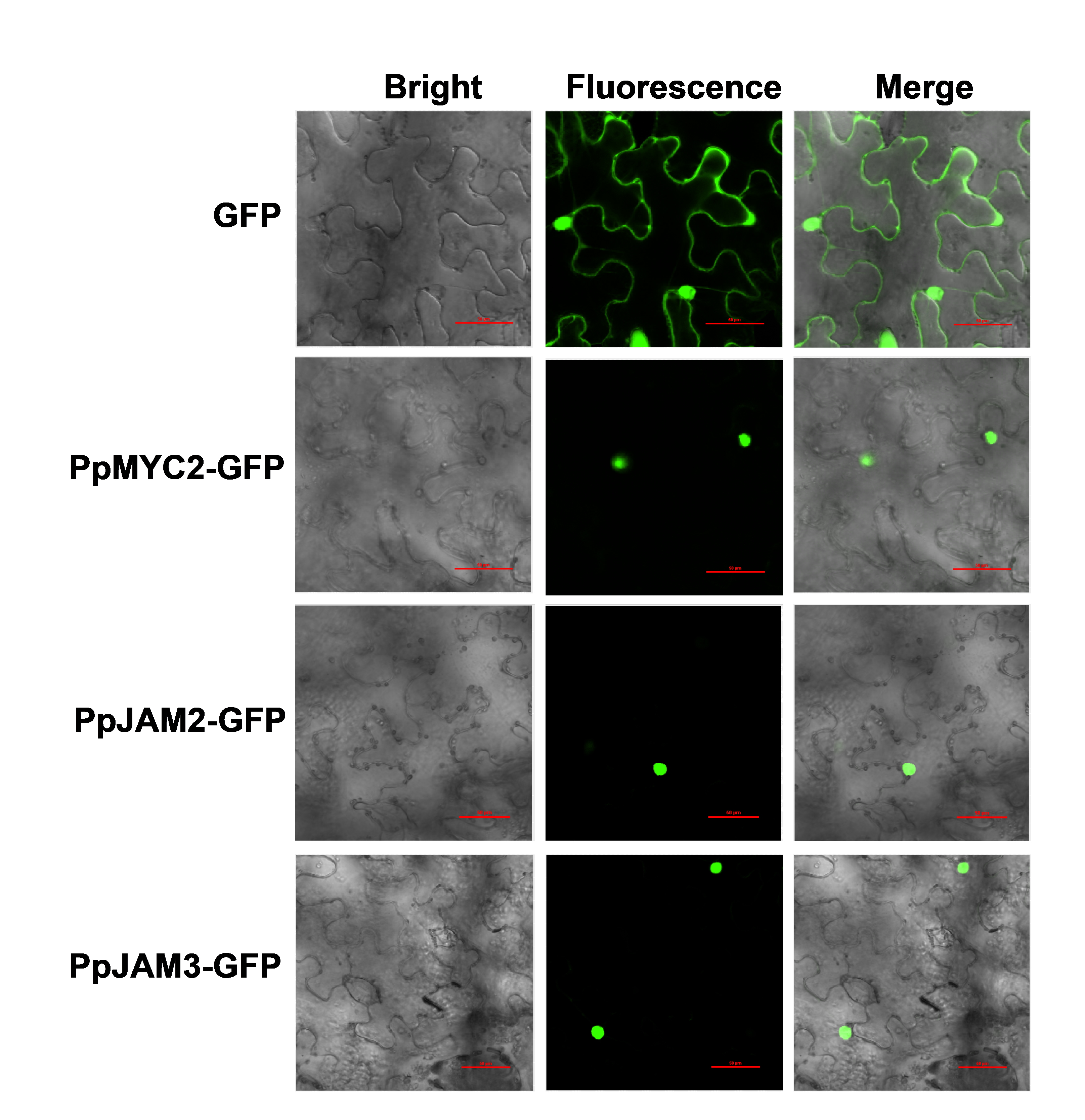


Figure S3. Subcellular localization of PpMYC2 and PpJAM2/3 in tobacco leaf cells. GFP signal was observed with a fluorescence microscope at 2 d after infiltration. Merge is the co-localization image; scale bar is 50 µm.

**Figure S4**

**
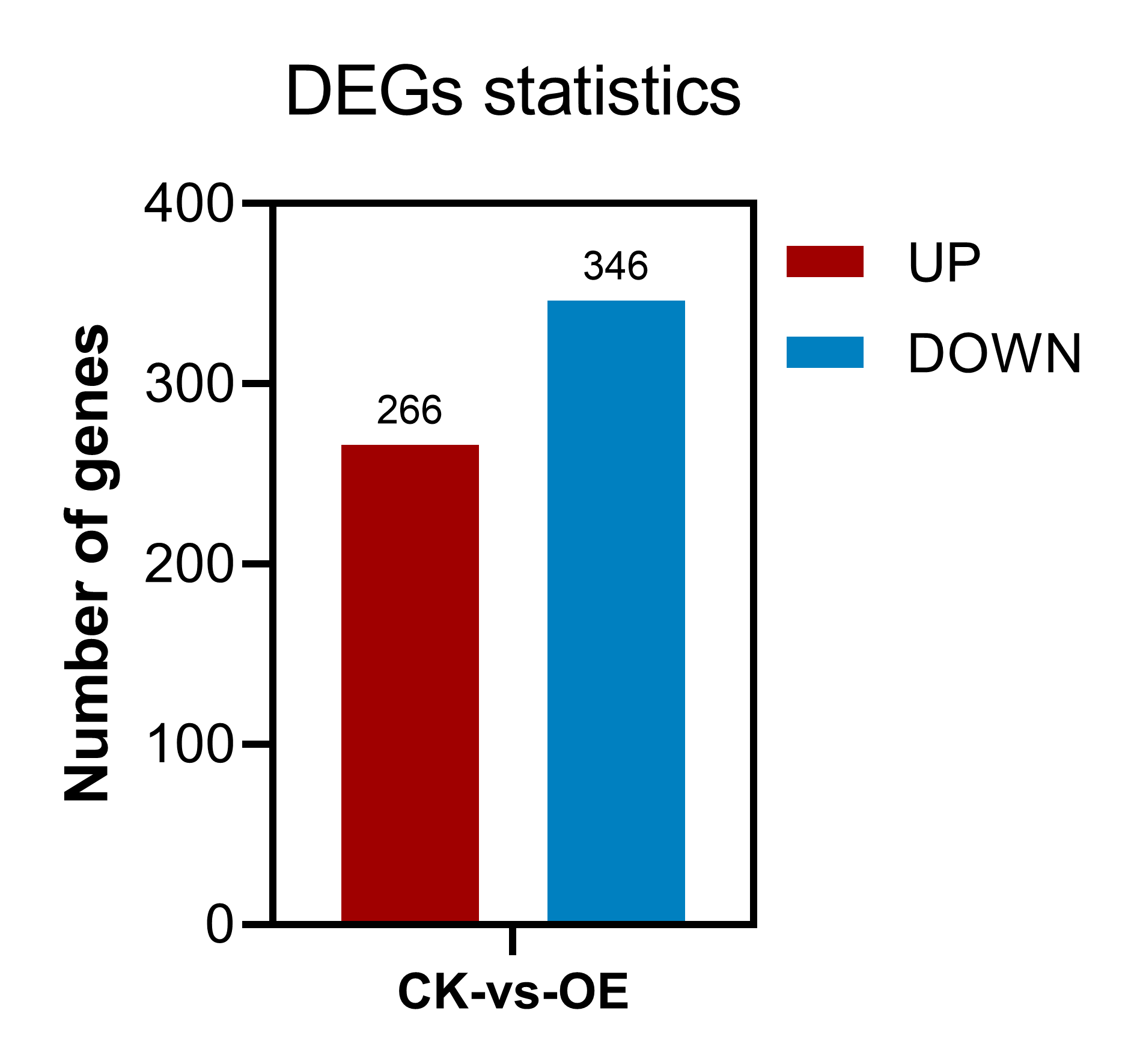
**

Figure S4. DEGs statistics. CK, the control peach fruit transformed with empty vector (pART-CAM); OE, peach fruit transformed with recombinant overexpression vector (pART-CAM-*PpMYC2*).
